# Enzymatic Synthesis of Modified *Alternaria* Mycotoxins Using a Whole-Cell Biotransformation System

**DOI:** 10.3390/toxins12040264

**Published:** 2020-04-20

**Authors:** Sophie Scheibenzuber, Thomas Hoffmann, Isabelle Effenberger, Wilfried Schwab, Stefan Asam, Michael Rychlik

**Affiliations:** 1 Chair of Analytical Food Chemistry, Technical University of Munich, Maximus-von-Imhof Forum 2, 85354 Freising, Germany; sophie.scheibenzuber@tum.de (S.S.); stefan.asam@tum.de (S.A.); 2 Biotechnology of Natural Products, Technical University of Munich, Liesel-Beckmann-Straße 1, 85354 Freising, Germany; tom.hoffmann@tum.de (T.H.); wilfried.schwab@tum.de (W.S.); 3 4GENE, Lise-Meitner-Straße 30, 85354 Freising, Germany; isabelle.effenberger@4gene.de

**Keywords:** *Alternaria* spp., mycotoxins, modified mycotoxins, glucosyltransferase, enzymatic synthesis, biotransformation, alternariol, alternariol monomethyl ether

## Abstract

Reference standards for *Alternaria* mycotoxins are rarely available, especially the modified mycotoxins alternariol-3-glucoside (AOH-3-G), alternariol-9-glucoside (AOH-9-G), and alternariol monomethylether-3-glucoside (AME-3-G). To obtain these three glucosides as analytical standards for method development and method validation, alternariol and alternariol monomethylether were enzymatically glycosylated in a whole-cell biotransformation system using a glycosyltransferase from strawberry (*Fragaria x ananassa*), namely UGT71A44, expressed in *Escherichia coli* (*E. coli)*. The formed glucosides were isolated, purified, and structurally characterized. The exact amount of the isolated compounds was determined using high-performance liquid chromatography with UV-detection (HPLC-UV) and quantitative nuclear resonance spectroscopy (qNMR). This method has proved to be highly effective with biotransformation rates of 58% for AOH-3-G, 5% for AOH-9-G, and 24% for AME-3-G.

## 1. Introduction

**Figure 1:**
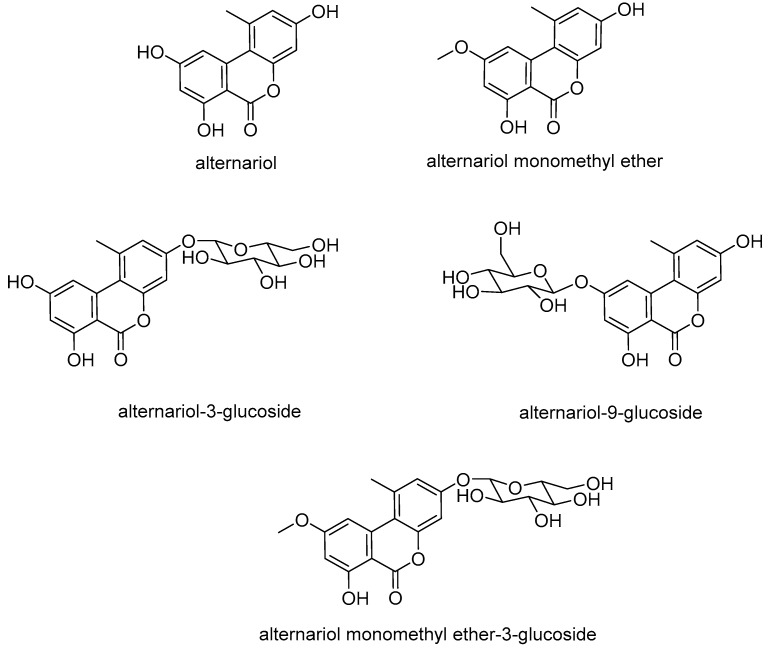
Chemical structures of dibenzopyrones alternariol (AOH), alternariol monomethyl ether (AME), and their respective glucosides.

Fungi of the genus *Alternaria* spp. are known to produce several structurally diverse mycotoxins, which are frequently detected in grain, vegetables, and fruits [1,2]. The most commonly known mycotoxins produced by these fungi are the dibenzopyrones alternariol (AOH), alternariol monomethyl ether (AME) (Figure 1), and altenuene, as well as the perylene quinones altertoxin I, altertoxin II, alterperylenol, and stemphyltoxin III, and the N-containing compounds tenuazonic acid (TeA) and tentoxin (TEN) [2].

The known toxicological impacts of these compounds are as diverse as their chemical structures. However, until now, most research was mainly conducted with the most abundant toxins AOH, AME, and TeA. Generally, no genotoxicity or carcinogenicity of *Alternaria* toxins could be proven in vivo, yet, several studies showed the genotoxic potential of AOH and AME in vitro in bacteria and mammalian cells [3,4,5,6].

Nevertheless, there are no regulations or legal limits for *Alternaria* toxins at this point due to the scarce availability of toxicity and occurrence data. Therefore, the European Food Safety Authority (EFSA) used the threshold of toxicological concern (TTC) approach to evaluate the relative risk of AOH, AME, TEN, and TeA in different population groups. Because of the in vitro genotoxicity of AOH and AME, the TTC value for these two mycotoxins was set to 2.5 ng/kg b.w. per day. For TeA and TEN, the TTC was defined as 1500 ng/kg b.w. per day as these two substances showed no genotoxic potential in mammalian cells [7]. With this definition, it was calculated from a dietary exposure study that AOH and AME are taken up in higher concentrations than expected, which might cause a risk to human health [8].

Furthermore, modified forms of AOH and AME have been brought into focus by analyses reported in the last few years. These compounds were either produced by the fungus, e.g., AOH-3-sulfate (AOH-3-S) and AME-3-sulfate (AME-3-S), detected in human cells during in vitro studies, e.g., AOH-3-glucuronide, AOH-9-glucuronide, and AME-3-glucuronide, or found in plants, e.g., AOH-3-glucoside (AOH-3-G), AOH-9-glucoside (AOH-9-G), and AME-3-glucoside (AME-3-G) [9,10,11] (Figure 1). 

Hence, the occurrence of sulfated and glycosylated AOH and AME in food commodities should be studied more intensively as modified mycotoxins might release their native precursor due to hydrolysis in the digestive tract, which elevates the total exposure towards AOH and AME. Until now, little data for modified *Alternaria* toxins in food products are available: Walravens et al. [12] analyzed different tomato products and found the sulfated forms of AOH and AME with values of up to 8.7 ug/kg (AOH-3-S) and 9.9 ug/kg (AME-3-S), while the glucosides were not detected at all. In addition, no modified *Alternaria* toxin could be found in rice and oat samples [13]. Furthermore, AOH-3-G and AOH-9-G were detected in one and two tomato products, respectively, while AOH-3-S and AME-3-S were present in two and five tomato products, respectively [14]. However, analytical methods for modified *Alternaria* toxins are scarce, and, therefore, information about their occurrence as well as their toxicological impact is lacking, and more research has to be conducted in this area. 

It has been shown [11] that glucosides of *Alternaria* toxins are not produced by the fungus itself. Thus, it has been hypothesized that these mycotoxins are glycosylated by the infected plant, most likely by enzymatic processes with the objective of detoxification. Within the research area of enzymes, glycosyltransferases (GTs) are an important group as they catalyze the transfer of sugar moieties from activated donors (e.g., uridine diphosphate glucose; UDP-glucose) to specific acceptor molecules (e.g., alcohols or carboxylic acids) which results in *O*-glucosides and glucose esters. However, the formation of *N*-, *S*-, and *C*-glycosides is also possible [15,16,17]. In biotechnology, the family 1 GTs have drawn special interest as they are able to catalyze the glycosylation of substrates both regio- and stereoselectively in high yield [18,19]. Using that knowledge, whole-cell biotransformation systems have been developed for the production of different compounds, e.g., glucosides of aroma active compounds, or large-scale syntheses of glucosides for industrial use in drug development, cosmetics, and functional foods [20,21]. Until now, the biocatalyst of choice for the whole-cell glycosylation of natural products is the well-studied bacterium *Escherichia coli* (*E. coli*), but usage of other biocatalysts, such as *Saccharomyces cerevisiae* or further yeasts, can also be found in literature [22,23,24]. When developing or optimizing biotransformation systems, it is economically advantageous to use biocatalysts that work with an inexpensive sugar source, such as sucrose. In addition, an in-situ regeneration of UDP-glucose should be carried out by either adding sucrose synthases (SUS) or using biocatalysts that are able to express SUS themselves as high concentrations of UDP might have a negative impact on the glycosylation rate [25,26].

**Figure 2:**
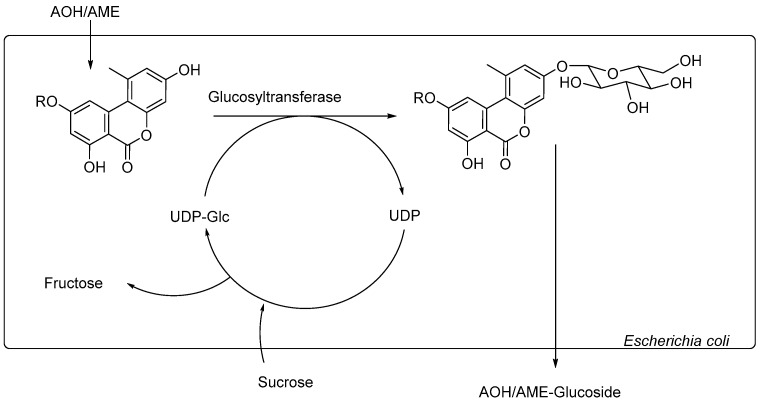
Schematic demonstration of the reactions within the biotransformation system. R = H for AOH, R = CH_3_ for AME. The arrows demonstrate the recycling of UDP-Glucose: Sucrose from the culture media reacts with UDP to UDP-Glc under the elimination of fructose.

With this information at hand, we aimed to develop a whole-cell biotransformation system using *E. coli* Waksman as a biocatalyst to enzymatically synthesize the modified *Alternaria* mycotoxins AOH-3-G, AOH-9-G, and AME-3-G (Figure 2).

## 2. Results and Discussion

For this study, both pure AOH and AME and a partly purified fungal extract containing AOH and AME were used. The enzyme screening, as well as one batch of the biotransformation, was conducted with an extract from a fungal rice culture that showed high concentrations of AOH and AME in preliminary high-performance liquid chromatography (HPLC) measurements and was accessible in reasonable amounts and with acceptable effort. However, to determine the absolute biotransformation rate of the enzymatic synthesis, another batch of biotransformation was also performed with pure compounds. From these results, the absolute biotransformation rate could be calculated, which was not possible from the batch using the fungal extract as the precise concentrations of AOH and AME could not be determined due to overlapping matrix substances in the HPLC analyses.

### 2.1. Screening for UGTs Converting AOH and AME

**Figure 3:**
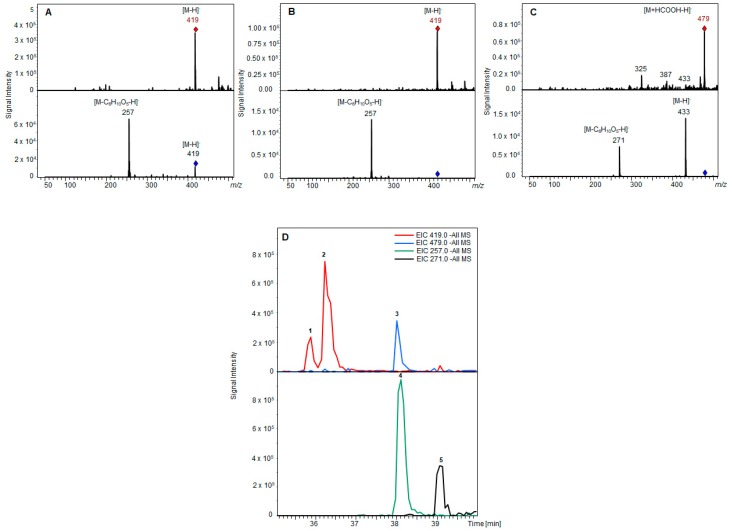
LC-MS analysis of mycotoxin glucosides. Full scan mass spectra (top) and product ion mass spectra (bottom) of alternariol glucoside isomers (**A**,**B**) and alternariol monomethyl ether glucoside (**C**), each showing the loss of one glucose residue (C_6_H_10_O_5_; red/blue rhombus: precursor ion markers). LC-MS extracted ion chromatograms (**D**) of products after biotransformation (alternariol glucoside isomers (1 and 2), alternariol monomethyl ether glucoside (3)) and substrates (alternariol (4), alternariol monomethyl ether (5)).

**Figure 4:**
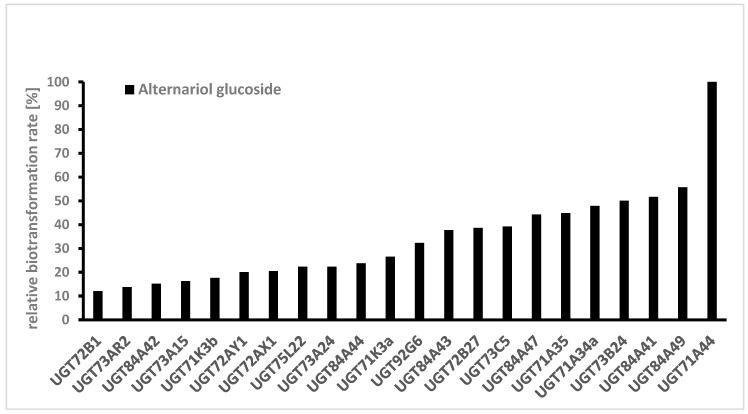
Screening of a UDP-glucosyltransferase (UGT) library consisting of 61 UGTs towards alternariol. Only UGTs with a relative biotransformation rate higher than 10% are shown. Glucoside formation was monitored by LC-MS, and the UGT with the highest value was set to 100% (single determinations).

Several UDP-glucosyltransferases (UGTs) expressed in *E. coli* were screened for activity towards AOH in a high-throughput whole-cell biotransformation system. The UGT library consisted of 61 enzymes from grapevine (*Vitis vinifera*), strawberry (*Fragaria* spp.), raspberry (*Rubus idaeus*), tobacco (*Nicotiana benthamiana*), Arabidopsis (*Arabidopsis thaliana*), tea (*Camellia sinensis*), peppermint (*Mentha x piperita*), Madagascar periwinkle (*Catharanthus roseus*), and a yeast (*Starmerella bombicola*). In a microscale approach, 5 mL cultures of respective *E. coli* W:pGEX-4T1:UGT clones were treated with a fungal extract containing a mixture of AOH and AME. After 2 days, the cultures were analyzed with LC-MS and the formed glucosides identified according to their mass spectrum (Figure 3). Relative biotransformation rates (combined value for AOH-3-G and AOH-9-G) were calculated and enzyme UGT71A44, a glucosyltransferase from strawberry (*Fragaria x ananassa*), previously known to produce quercetin 3- and 7-O-glucoside [27] was identified as the superior enzyme for producing AOH glucosides (Figure 4). Almost all of the aglycon was transformed by this enzyme. Out of the 61 UGTs tested, 22 showed relative biotransformation rates of more than 10%, while 7 showed more than 40% compared to UGT71A44 (100%).

**Figure 5:**
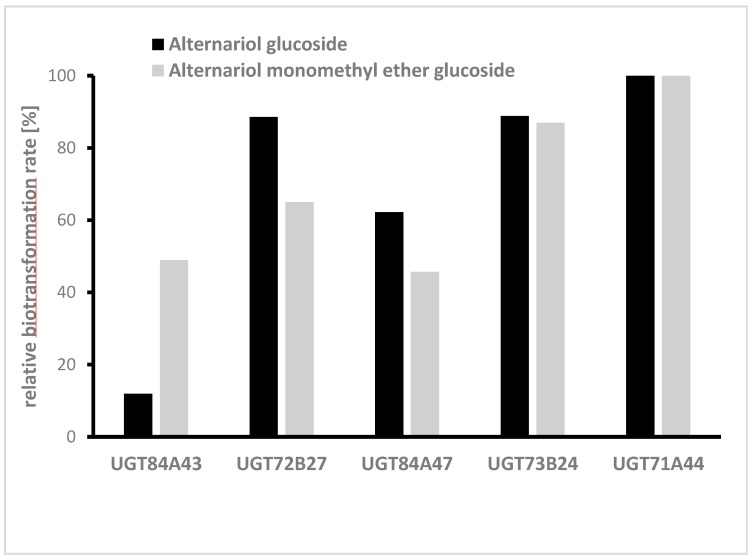
Relative biotransformation rate of five UGTs towards alternariol (AOH) and alternariol monomethyl ether (AME) (single determinations). UGTs were selected because they showed a high biotransformation rate in the initial screening.

In the next step, glycosylation of AOH and AME was studied separately in medium-scale cultures, also using the fungal extract. Therefore, five UGTs (UGT84A43, UGT72B27, UGT84A47, UGT73B24, and UGT71A44) already identified in the initial screening were chosen, and the biotransformation of the aglycons was monitored in 50 mL cultures. All 5 UGTs produced glucosides of AOH and AME, but the highest transformation rates for both mycotoxins were UGT71A44 (Figure 5).

Therefore, for the final step, i.e., the biotransformation in large scale cultures and the determination of the absolute biotransformation rate, 100 mL *E. coli* W:pGEX-4T^:UGT71A44 cultures were employed to produce AOH glucosides and AME glucoside by transforming pure AOH and AME, respectively.

### 2.2. Isolation and Purification of AOH-3-G, AOH-9-G, and AME-3-G

After biotransformation, the sterile filtered medium was directly mixed with the polystyrenic non-ionic adsorbent resin Purosorb^TM^ PAD600 to bind the desired analytes. Using vacuum filtration, the solid phase was washed with water to remove remaining sugars, and then the glycosides were eluted with methanol. As reduced stability of AOH-9-G in MeOH was expected [28], the eluate was evaporated immediately and taken up in a mixture of ACN/H_2_O (3:2). Due to the presence of a yellow colorant, it was necessary to perform a two-step semi-preparative HPLC separation to isolate and purify the target compounds. First, an ACN/H_2_O gradient (method 1) with a flow rate of 1 mL/min was used, then the collected fractions were further purified using a MeOH/H_2_O gradient (method 2) with a flow rate of 0.8 mL/min.

**Figure 6:**
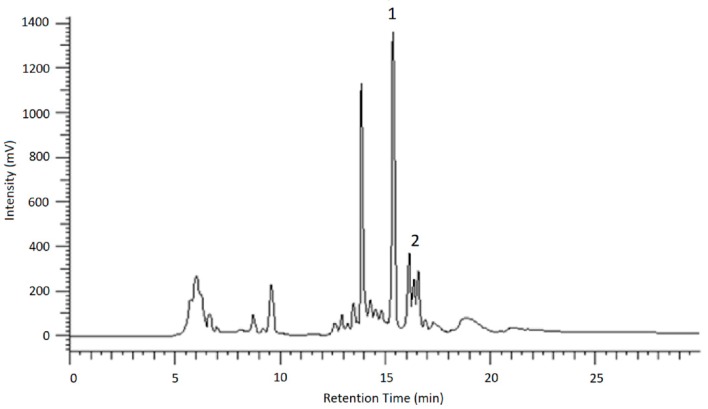
HPLC-UV chromatogram showing the separation of AOH-glucosides (1) and AME-glucoside (2) using method 1.

**Figure 7:**
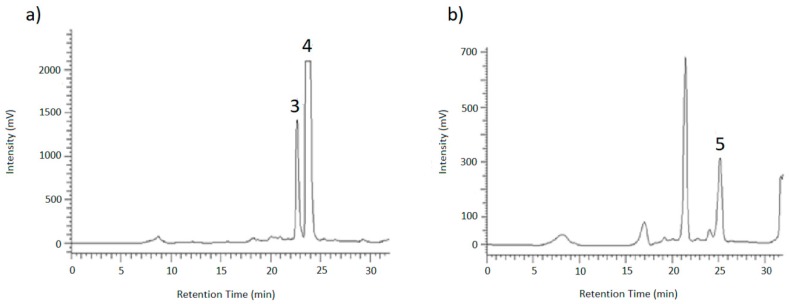
HPLC-UV chromatograms of method 2 showing (**a**) the separation of AOH-9-G (3) and AOH-3-G (4) and (**b**) the purification of AME-3-G (5).

To identify the resulting peaks and hence, the retention time of the desired analytes, eluates of the chromatographic runs shown in Figure 6 and Figure 7 were collected in vials for 1 min each, dried under nitrogen and then taken up in ACN/H_2_O (3/7; *v*/*v*) for LC-MS and HPLC measurements. After the desired analytes were assigned to their corresponding signals using reference compounds, the glucosides were purified in this way.

### 2.3. Structural Identification

**Figure 8:**
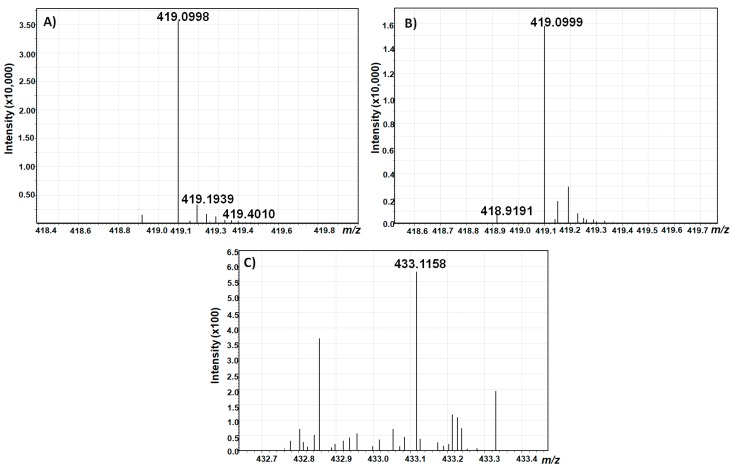
LC-Q-TOF-MS Scans of AOH-3-G (**A**), AOH-9-G (**B**), and AME-3-G (**C**).

To further verify the identity of the collected compounds, a high resolution scan was performed using a hybrid quadrupole time-of-flight mass spectrometer coupled to liquid chromatography (LC-Q-TOF-MS) in the negative electrospray ionization (ESI) mode (Figure 8). 

With a calculated theoretical mass of [M–H]^−^ = 419,0983736 g/mol for AOH-3-G and AOH-9-G, and [M–H]^−^ = 433,1140236 g/mol for AME-3-G, the mass accuracy of the measurements was below 4 ppm for both alternariol glucosides, and below 5 ppm for AME glucoside, which confirms the identity of the isolated toxins.

However, the full-scan spectrum revealed that AME-3-G could not be completely purified, but, as the measurement showed the absence of any other known *Alternaria* toxin in the collected fraction, it was decided not to further purify this compound due to unavoidable losses during purification.

### 2.4. Quantification and Determination of Biotransformation Rate

All obtained glucosides were quantified using HPLC-UV with external calibration. For this purpose, respective reference compounds of AOH-3-G, AOH-9-G, and AME-9-G were used that were supplied by Hannes Puntscher, University of Vienna. Additionally, AOH-3-G and AOH-9-G were obtained in suitable purity and amounts to determine the concentration also using quantitative NMR (qNMR) techniques. However, this was not possible for AME-3-G due to interfering impurities that deteriorated the accuracy of the qNMR measurement.

#### 2.4.1. qNMR Measurements of AOH-3-G and AOH-9-G

^1^H-NMR spectra were measured for both AOH-3-G and AOH-9-G, which showed no impurities despite formic acid remaining from the mobile phase of the semi-preparative HPLC. Signals were assigned according to literature and then integrated for quantitative analysis. Using L-tyrosine as a reference substance, at least three signals from each analyte were chosen for quantification (Table 1).

#### 2.4.2. Quantification of AOH-3-G, AOH-9-G, and AME-3-G from Fungal Extracts

Quantification of AOH-3-G and AOH-9-G with qNMR resulted in absolute amounts of 231.1 µg and 17.8 µg, respectively. These results confirmed the first impression from peak intensities during semi-preparative HPLC analysis that the enzyme preferably formed AOH-3-G and much less AOH-9-G. AME-3-G was quantified using HPLC-UV with external calibration and was obtained in amounts of 11.6 µg out of the fungal extract.

#### 2.4.3. Determination of UV Molar Extinction Coefficients for AOH-3-G and AOH-9-G

Additionally, absorption maxima and UV molar extinction coefficients of AOH-3-G and AOH-9-G were determined by spectrophotometric measurements to characterize those almost unknown modified mycotoxins further. As expected, both analytes showed nearly identical absorption maxima, i.e., 255 nm and 338 nm for AOH-3-G, and 254 nm and 338 nm for AOH-9-G. Extinction coefficients for both analytes were determined in acetonitrile using three different concentrations (1, 2.5 and 5 µg/mL) in triplicate resulting in mean ε_255nm_ = 47,600 ± 500 L/cm·mol for AOH-3-G and ε_254nm_ = 54,900 ± 600 L/cm·mol for AOH-9-G.

#### 2.4.4. Absolute Biotransformation Rate of AOH and AME

AOH-3-G, AOH-9-G, and AME-3-G obtained by biotransformation from pure AOH and AME were quantified using HPLC-UV and an external three-point calibration. Hence, quantitative analysis revealed that out of 288 µg AOH used for biotransformation, 160 µg AOH-3-G and 15 µg AOH-9-G were obtained, which results in an absolute biotransformation rate of 58% for AOH-3-G and 5% for AOH-9-G and, therefore, a total absolute biotransformation rate of 63% for AOH glucosides. With 36 µg AME-3-G obtained out of 150 µg AME, the absolute biotransformation rate of this compound was 24%.

### 2.5. Obtained Amounts of Glucosides

Taken together, from the two large scale cultures, absolute amounts of 391.1 µg AOH-3-G, 32.8 µg AOH-9-G, and 47.5 µg AME-3-G were obtained as pure compounds.

## 3. Conclusions

The whole-cell biotransformation using *E. coli* Waksman bacteria was successfully used to synthesize the modified *Alternaria* toxins AOH-3-glucoside, AOH-9-glucoside, and AME-3-glucoside. The obtained analytes could be purified by semipreparative HPLC and quantified using qNMR and HPLC-UV. Besides biotransformation using pure compounds, both AOH and AME could also be successively transformed into their glucosides in a partially purified fungal extract, which is a more economical approach. 

Additionally, quantitative data showed the preferred formation of AOH-3-G over AOH-9-G for UGT71A44, while AME-3-G was synthesized in similar yields to AOH-9-G. To increase the yield of AOH-9-G and AME-3-G, enzyme screening could be optimized towards the preferred formation of these two compounds. In the present study, the ratio of AOH-3-G and AOH-9-G was not determined in the first screening, and only enzymes that formed AOH-glucosides were tested for their activity towards AME. However, with a total biotransformation rate of 63% AOH-glucosides and 23% AME-glucoside, this method is a good alternative to a total chemical synthesis, where the final yield over all synthetical steps was reported to be only 0.1% or less [28].

Since one-third of the GTs tested were capable of glucosylating the mycotoxins, numerous plants are obviously capable of producing AOH glucosides, which have so far escaped analysis.

## 4. Materials and Methods

### 4.1. Starting Materials

The fungal extract was obtained by cultivating *Alternaria alternata* on rice for 7 days at 28 °C in a water shaking bath, grinding the moldy rice thoroughly, and extracting it three times with dichloromethane and methanol. After purification over silica, the fractions containing AOH and AME were combined, evaporated, and resolved in acetonitrile. To obtain pure AOH and AME, the respective fractions were further purified using HPLC-UV (La Chrom, D-7000, Merck, Germany in cooperation with Hitachi Instruments Inc., San Jose, CA, USA) [29]. Reference standards for AOH-3-G, AOH-9-G, and AME-3-G for LC-MS/MS and HPLC measurements were provided by Hannes Puntscher, Department of Food Chemistry and Toxicology, University of Vienna.

### 4.2. UGT-Library

61 UGT genes from Vitis vinifera, Fragaria × ananassa, Fragaria vesca, Camellia sinensis, Mentha × piperita, Starmerella bombicola, Nicotiana benthamiana, Arabidopsis thaliana, Catharanthus roseus, and Rubus idaeus were cloned into pGEX-4T1 and transferred into Escherichia coli W [30] resulting in a glucosyltransferase library (UGT88A23, UGT73E14, UGT88A26, UGT85A69co, UGT75L24, UGT75T1, UGT76Q3, UGT88A24, UGT85K11, UGT88F12, UGT73E13, UGT76Q2, UGT86C10, UGT88A12, UGT88A25, UGT708M2, UGT88A27, UGT88A21, UGT88A27, UGT73E12, UGT71W2, UGT709C6, UGT76Q4, UGT73E16, UGT730A1, UGT73E15, UGT85A70, UGT84A48, UGT75L23, UGT73B23, UGT709C7, UGT71AJ1, UGT85A69, UGT88A22, UGT88A20, UGT709C8, UGT85A72, UGT71A33, UGT84A46, UGT84A45, UGT72B1, UGT73AR2, UGT84A42, UGT73A15, UGT71K3b, UGT72AY1, UGT72AX1, UGT75L22, UGT73A24, UGT84A44, UGT71K3a, UGT92G6, UGT84A43, UGT72B27, UGT73C5, UGT84A47, UGT71A35, UGT71A34a, UGT73B24, UGT84A41, UGT84A49, UGT71A44; http://prime.vetmed.wsu.edu/resources/udp-glucuronsyltransferase-hompage)

### 4.3. Screening of UGTs

The screening of the UGT-library was carried out, as described previously [25]. AOH and AME were added as a mixture at a final concentration of approximately 1 mg/L and 0.2 mg/L, respectively. Glucoside formation was analyzed by LC-MS applying electrospray ionization in the negative mode. For relative quantification peak areas in extracted ion chromatograms, *m/z* 419 (AOH glucoside isomers, [M–H]^−^) and *m/z* 479 (AME glucoside, formate adduct, [M–H]^−^) were used.

### 4.4. LC-MS and LC-Q-TOF Measurements

An Agilent 6320 Ion Trap spectrometer equipped with an Agilent 1100 HPLC-DAD system (Agilent Tech. Inc., Santa Clara, CA, USA) was used for glucoside identification after the enzymatic screening. Glucoside separation was performed with a LUNA 3 µm, C18(2), 100 Å, 150 × 2 mm column (Phenomenex, Aschaffenburg, Germany). The gradient program was as follows: 0–30 min 0%–50% B, 30–35 min 50%–100% B, 35–50 min 100% B, 50–55 min 100%–0% B, 55–56 min 0% B with a flow rate of 0.2 mL/min. Solvent A was 0.1% formic acid in water, solvent B methanol with 0.1% formic acid. The injection volume was 5 µL. The ESI voltage of the capillary was set to +4000 V, and the endplate to −500 V. The drying gas was nitrogen at a temperature of 330 °C and a flow rate of 9 L/min. The full scan mass spectra were measured in a scan range from 50 to 975 *m/z* with a scan resolution of 26,000 m/z/s until the Ion charge Control reached 10,000 or 200 ms, whichever was achieved first. Tandem MS was carried out using helium as the collision gas (4 × 10^−6^ mbar) with 1 V collision voltage. Spectra were acquired in negative ionization mode. Software for data analysis was DataAnalysis 4.0 (build 234) (Bruker Daltonics, Bremen, Germany).

For the performance of the high-resolution measurements, a Nexera LC-40 System (Shimadzu, Kyoto, Japan) was coupled with an LCMS-9030 Q-TOF (Shimadzu, Kyoto, Japan) using the negative ESI mode. Scans were performed with ion accumulation (ID off) by direct injection of 1–4 µL of the analyte, using an isocratic flow of ACN/water (70/30) with a flow rate of 0.3 mL/min. Parameters at the LCMS-9030 were as follows: the nebulizing gas flow was 3 L/min, the heating gas flow was 10 L/min, and the drying gas flow was 10 L/min. The interface temperature was set to 300 °C, the heat block temperature to 400 °C, and the desolvation temperature was set to 250 °C; the interface voltage was 4 kV. External mass calibration using clusters of NaI was performed. For data analysis, the LabSolution software (Shimadzu, Kyoto, Japan) was used.

### 4.5. Whole-Cell Biotransformation on Large Scale

Large scale glucoside production was done according to a published procedure [25] in 400 mL shaking flasks containing 100 mL media. UGT expression was induced by isopropyl β-D-1-thiogalactopyranoside (0.2 mM, OD600 1.5), and biotransformation was started by feeding the substrates. Final aglyca concentrations were 288 µg/L AOH and 150 µg/L AME, respectively. After 48 h of incubation at 18 °C, the bacterial cells were removed by centrifugation. The sterile filtered supernatant (Polyether sulfone membrane filter 0.22 µm, Filtropur V50, Sarstedt, Nümbrecht, Germany) was used for product isolation.

### 4.6. Isolation of the Analytes after Biotransformation

An adequate amount, i.e., 70–80 g, of Purosorb^TM^ PAD600 solution was added to the sterile filtered culture medium. The mixture was then incubated on a horizontal shaker at 250 rpm overnight. The suspension was vacuum filtrated (qualitative filter paper 413, 5–13 µm, VWR International, Leuven, Belgium), and the resin was washed with 5 volumes of water. Then, the glucosides were eluted by filtering 10 volumes of methanol over the resin. Afterward, the eluate was evaporated at 30 °C using a rotary evaporator. All fractions, i.e., the filtrate, the washing steps, and the eluate, were collected separately and checked for AOH-3-G, AOH-9-G, and AME-3-G using LC-MS/MS.

### 4.7. Semi-Preparative HPLC

A Merck Hitachi HPLC System (LaChrom, D-7000, Merck, Germany, in cooperation with Hitachi Instruments Inc., San Jose, CA, USA) was used to isolate and purify the three different glucosides. Separation was performed with a Pro-Pack C18 150 × 10.0 mm, S-5 µm, 12 nm column (YMC, YMC Europe GmbH, Dienslaken, Germany) at room temperature. Detection was conducted with a UV detector (L-7400) at 254 nm. The gradient program for method 1 (separation of AOH-G isomers and AME-G) was as follows: 0–3 min 10% B, 3–23 min 10%–100% B, 23–25 min 100% B, 25–30 min 100%–10% B, 30–35 10% B with a flow rate of 1 mL/min. Solvent A was water, and solvent B was acetonitrile. For method 2 (separation of AOH-3-G and AOH-9-G and purification of AME-3-G) the gradient was: 0–5 min 50% B, 5–7 min 50%–65% B, 7–19 min 65% B, 19–21 min 65%–100% B, 21–24 min 100% B, 24–27 min 100%–50% B, 27–32 min 50% B with a flow rate of 0.8 mL/min. Solvent A was water, and solvent B was methanol. Software for data analysis was the HPLC system manager (LaChrom, Merck, Germany, in cooperation with Hitachi Instruments Inc., USA, version 4.1).

### 4.8. qNMR

^1^H-NMR measurements were conducted on a Bruker AVIII system (500 MHz, Bruker, Rheinstetten, Germany). Analytes were dissolved in deuterated methanol (Sigma–Aldrich, Steinheim, Germany) and filled into NMR tubes (5 × 178 mm, USC-tubes, Bruker, Faellanden, Switzerland). The final NMR settings were adjusted as described in the literature [31]. For quantitative NMR measurements, an L-tyrosine standard was measured in different concentrations beforehand; for data analysis, the TopSpin-3.6.0 Software (Bruker, Rheinstetten, Germany) was used with the ERETIC II function for quantification.

### 4.9. UV Spectroscopy

A Genesys 10S UV-Vis spectrophotometer (Thermo Fisher Scientific, Madison, WI, USA) was used with precision cells made of quartz glass (Hellma GmbH & Co. KG, Müllheim, Germany) for the characterization of the obtained analytes. Absorption maxima were determined by conducting a scanning run between 200 and 400 nm against acetonitrile with 1 µg/mL solutions of the glucosides. To obtain the extinction coefficients, the analytes were diluted to solutions of 1 µg/mL, 2.5 µg/mL, and 5 µg/mL, each measured in triplicates.

## Figures and Tables

**Table 1:** NMR Data for alternariol-3-glucoside (AOH-3-G) and alternariol-9-glucoside (AOH-9-G).

Analyte	δ ppm	Integral	Multiplicity	J (Hz)	qNMR (mmol/600 µL)
AOH-3-G	7.31	1H	d	2.1	-
	6.99	1H	d	2.7	0.924
	6.97	1H	d	2.7	0.917
	6.41	1H	d	2.1	-
	3.95–3.90	1H	m	-	0.919
	3.74–3.67	1H	m	-	-
	3.55–3.50	1H	m	-	-
	3.42–3.37	1H	m	-	-
	2.82	3H	s	-	-
AOH-9-G	7.51	1H	d	2.2	0.070
	6.69	1H	d	2.7	0.068
	6.67	1H	d	2.7	-
	6.59	1H	d	2.2	0.065
	3.96–3.90	1H	m	-	0.066
	3.77–3.70	1H	m	-	-
	3.68–3.62	2H	m	-	-
	3.6–3.56	1H	m	-	-
	2.81	3H	s	-	-

-, data not available.
